# 2-Methyl-1-(4-methyl­phenyl­sulfon­yl)naphtho­[2,1-*b*]furan

**DOI:** 10.1107/S1600536812012159

**Published:** 2012-03-28

**Authors:** Hong Dae Choi, Pil Ja Seo, Uk Lee

**Affiliations:** a Department of Chemistry, Dongeui University, San 24 Kaya-dong Busanjin-gu, Busan 614-714, Republic of Korea; b Department of Chemistry, Pukyong National University, 599-1 Daeyeon 3-dong, Nam-gu, Busan 608-737, Republic of Korea

## Abstract

In the title compound, C_20_H_16_O_3_S, the 4-methyl­phenyl ring makes a dihedral angle of 83.07 (3)° with the mean plane [r.m.s. deviation = 0.020 (1) Å] of the naphtho­furan fragment. In the crystal, mol­ecules are linked by weak C—H⋯O and C—H⋯π inter­actions.

## Related literature

For background information and the crystal structures of related compounds, see: Choi *et al.* (2008 ▶, 2012 ▶).
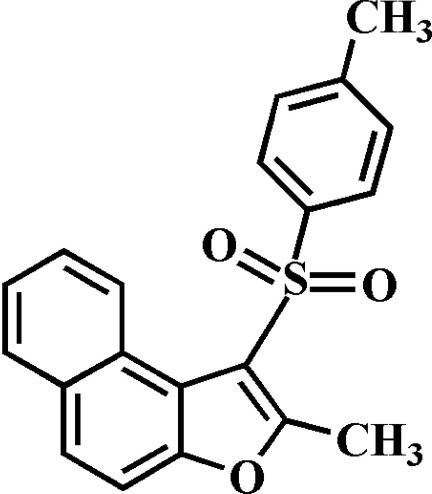


## Experimental

### 

#### Crystal data

C_20_H_16_O_3_S
*M* *_r_* = 336.39
Monoclinic, 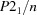

*a* = 8.6628 (2) Å
*b* = 6.3669 (1) Å
*c* = 28.9119 (6) Å
β = 96.795 (1)°
*V* = 1583.44 (6) Å^3^
*Z* = 4
Mo *K*α radiation
μ = 0.22 mm^−1^
*T* = 173 K
0.35 × 0.32 × 0.28 mm

#### Data collection

Bruker SMART APEXII CCD diffractometer
Absorption correction: multi-scan (*SADABS*; Bruker, 2009 ▶) *T*_min_ = 0.676, *T*_max_ = 0.746
14714 measured reflections
3958 independent reflections
3363 reflections with *I* > 2σ(*I*)
*R*_int_ = 0.029

#### Refinement

*R*[*F* ^2^ > 2σ(*F* ^2^)] = 0.040
*wR*(*F* ^2^) = 0.113
*S* = 1.04
3958 reflections
219 parameters
H-atom parameters constrained
Δρ_max_ = 0.32 e Å^−3^
Δρ_min_ = −0.49 e Å^−3^

### 

Data collection: *APEX2* (Bruker, 2009 ▶); cell refinement: *SAINT* (Bruker, 2009 ▶); data reduction: *SAINT*; program(s) used to solve structure: *SHELXS97* (Sheldrick, 2008 ▶); program(s) used to refine structure: *SHELXL97* (Sheldrick, 2008 ▶); molecular graphics: *ORTEP-3* (Farrugia, 1997 ▶) and *DIAMOND* (Brandenburg, 1998 ▶); software used to prepare material for publication: *SHELXL97*.

## Supplementary Material

Crystal structure: contains datablock(s) global, I. DOI: 10.1107/S1600536812012159/hb6696sup1.cif

Structure factors: contains datablock(s) I. DOI: 10.1107/S1600536812012159/hb6696Isup2.hkl

Supplementary material file. DOI: 10.1107/S1600536812012159/hb6696Isup3.cml

Additional supplementary materials:  crystallographic information; 3D view; checkCIF report

## Figures and Tables

**Table 1:** Hydrogen-bond geometry (Å, °) *Cg* is the centroid of the C14–C19 4-methyl­phenyl ring.

*D*—H⋯*A*	*D*—H	H⋯*A*	*D*⋯*A*	*D*—H⋯*A*
C13—H13*A*⋯O1^i^	0.98	2.54	3.503 (2)	168
C13—H13*C*⋯O3^ii^	0.98	2.49	3.440 (2)	164
C5—H5⋯*Cg*^iii^	0.95	2.80	3.648 (2)	149

Symmetry codes: (i) 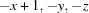; (ii) 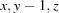; (iii) 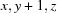.

**Fig. 1.**
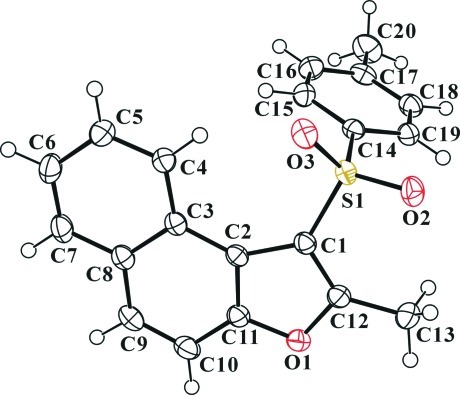
The molecular structure of the title compound with displacement ellipsoids drawn at the 50% probability level. H atoms are presented as small spheres of arbitrary radius.

**Fig. 2.**
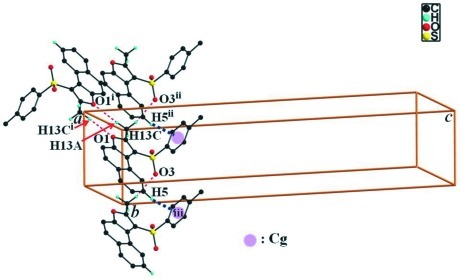
A view of the C—H···O and C—H···π interactions (dotted lines) in the crystal structure of the title compound. H atoms non-participating in hydrogen-bonding were omitted for clarity. [Symmetry codes: (i) -x + 1, - y, - z; (ii) x, y - 1, z; (iii) x, y + 1, z.]

## supplementary crystallographic information

### Comment

As a part of our ongoing study of 2-methylnaphtho[2,1-b]furan derivatives containing 1-phenylsulfonyl (Choi *et al.*, 2008) and 1-(4-methylphenylsulfinyl) (Choi *et al.*, 2012) substituents, we report herein the crystal structure of the title compound.

In the title molecule (Fig. 1), the naphthofuran unit is essentially planar, with a mean deviation of 0.020 (1) Å from the least-squares plane defined by the thirteen constituent atoms. The dihedral angle between the 4-methylphenyl ring and the mean plane of the naphthofuran fragment is 83.07 (3)°. The crystal packing features weak C—H···O hydrogen bonds (Fig. 2 & Table 1). In addition, weak C—H···π interactions occur (Fig. 2 & Table 1).

### Experimental

77% 3-Chloroperoxybenzoic acid (448 mg, 2.0 mmol) was added in small portions to a stirred solution of 2-methyl-1-(4-methylphenylsulfanyl)naphtho [2,1-b]furan (273 mg, 0.9 mmol) in dichloromethane (40 mL) at 273 K. After being stirred at room temperature for 10h, the mixture was washed with saturated sodium bicarbonate solution and the organic layer was separated, dried over magnesium sulfate, filtered and concentrated at reduced pressure. The residue was purified by column chromatography (benzene) to afford the title compound as a colorless solid [yield 68%, m.p. 440–441 K; *R*_f_ = 0.41 (benzene)]. Colourless blocks were prepared by slow evaporation of a solution of the title compound in acetone at room temperature.

### Refinement

All H atoms were positioned geometrically and refined using a riding model, with C—H = 0.95 Å for aryl and 0.98 Å for methyl H atoms. Uiso(H) = 1.2*U*_eq_(C) for aryl and 1.5*U*_eq_(C) for methyl H atoms. The positions of methyl hydrogens were optimized rotationally.

### Crystal data

**Table:**

C_20_H_16_O_3_S	*F*(000) = 704
*M**_r_* = 336.39	*D*_x_ = 1.411 Mg m^−^^3^
Monoclinic, *P*2_1_/*n*	Mo *K*α radiation, λ = 0.71073 Å
Hall symbol: -P 2yn	Cell parameters from 5369 reflections
*a* = 8.6628 (2) Å	θ = 2.4–28.2°
*b* = 6.3669 (1) Å	µ = 0.22 mm^−^^1^
*c* = 28.9119 (6) Å	*T* = 173 K
β = 96.795 (1)°	Block, colourless
*V* = 1583.44 (6) Å^3^	0.35 × 0.32 × 0.28 mm
*Z* = 4	

### Data collection

**Table:**

Bruker SMART APEXII CCD diffractometer	3958 independent reflections
Radiation source: rotating anode	3363 reflections with *I* > 2σ(*I*)
Graphite multilayer monochromator	*R*_int_ = 0.029
Detector resolution: 10.0 pixels mm^-1^	θ_max_ = 28.3°, θ_min_ = 1.4°
φ and ω scans	*h* = −11→11
Absorption correction: multi-scan (*SADABS*; Bruker, 2009)	*k* = −8→8
*T*_min_ = 0.676, *T*_max_ = 0.746	*l* = −38→36
14714 measured reflections	

### Refinement

**Table:**

Refinement on *F*^2^	Primary atom site location: structure-invariant direct methods
Least-squares matrix: full	Secondary atom site location: difference Fourier map
*R*[*F*^2^ > 2σ(*F*^2^)] = 0.040	Hydrogen site location: difference Fourier map
*wR*(*F*^2^) = 0.113	H-atom parameters constrained
*S* = 1.04	*w* = 1/[σ^2^(*F*_o_^2^) + (0.0592*P*)^2^ + 0.6896*P*] where *P* = (*F*_o_^2^ + 2*F*_c_^2^)/3
3958 reflections	(Δ/σ)_max_ < 0.001
219 parameters	Δρ_max_ = 0.32 e Å^−^^3^
0 restraints	Δρ_min_ = −0.49 e Å^−^^3^

### Special details

**Table:**

Geometry. All esds (except the esd in the dihedral angle between two l.s. planes) are estimated using the full covariance matrix. The cell esds are taken into account individually in the estimation of esds in distances, angles and torsion angles; correlations between esds in cell parameters are only used when they are defined by crystal symmetry. An approximate (isotropic) treatment of cell esds is used for estimating esds involving l.s. planes.
Refinement. Refinement of F^2^ against ALL reflections. The weighted R-factor wR and goodness of fit S are based on F^2^, conventional R-factors R are based on F, with F set to zero for negative F^2^. The threshold expression of F^2^ > 2sigma(F^2^) is used only for calculating R-factors(gt) etc. and is not relevant to the choice of reflections for refinement. R-factors based on F^2^ are statistically about twice as large as those based on F, and R- factors based on ALL data will be even larger.

### Fractional atomic coordinates and isotropic or equivalent isotropic displacement parameters (Å^2^)

**Table:**

	*x*	*y*	*z*	*U*_iso_*/*U*_eq_	
S1	0.27965 (4)	0.47230 (6)	0.122492 (12)	0.02209 (11)	
O1	0.53486 (13)	0.23737 (18)	0.03339 (4)	0.0289 (3)	
O2	0.14170 (12)	0.35873 (19)	0.10517 (4)	0.0307 (3)	
O3	0.26753 (12)	0.69453 (18)	0.12988 (4)	0.0298 (3)	
C1	0.41907 (16)	0.4236 (2)	0.08492 (5)	0.0219 (3)	
C2	0.56531 (16)	0.5295 (2)	0.07891 (5)	0.0213 (3)	
C3	0.65037 (16)	0.7128 (2)	0.09593 (5)	0.0218 (3)	
C4	0.60027 (17)	0.8614 (2)	0.12726 (5)	0.0247 (3)	
H4	0.5033	0.8415	0.1389	0.030*	
C5	0.68864 (19)	1.0335 (3)	0.14119 (6)	0.0295 (3)	
H5	0.6519	1.1313	0.1622	0.035*	
C6	0.83300 (19)	1.0672 (3)	0.12478 (6)	0.0332 (4)	
H6	0.8936	1.1870	0.1345	0.040*	
C7	0.88506 (18)	0.9260 (3)	0.09476 (6)	0.0316 (4)	
H7	0.9831	0.9485	0.0840	0.038*	
C8	0.79719 (16)	0.7470 (2)	0.07925 (5)	0.0254 (3)	
C9	0.85486 (18)	0.6061 (3)	0.04712 (6)	0.0305 (3)	
H9	0.9540	0.6316	0.0373	0.037*	
C10	0.77213 (19)	0.4364 (3)	0.03018 (6)	0.0308 (3)	
H10	0.8093	0.3442	0.0082	0.037*	
C11	0.62874 (18)	0.4049 (2)	0.04693 (5)	0.0251 (3)	
C12	0.40872 (18)	0.2496 (2)	0.05693 (5)	0.0260 (3)	
C13	0.2966 (2)	0.0750 (3)	0.04686 (6)	0.0349 (4)	
H13A	0.3285	−0.0119	0.0217	0.052*	
H13B	0.1927	0.1325	0.0374	0.052*	
H13C	0.2943	−0.0110	0.0749	0.052*	
C14	0.35605 (16)	0.3550 (2)	0.17546 (5)	0.0218 (3)	
C15	0.46746 (18)	0.4594 (3)	0.20557 (5)	0.0279 (3)	
H15	0.4992	0.5975	0.1985	0.033*	
C16	0.53175 (19)	0.3600 (3)	0.24596 (5)	0.0320 (4)	
H16	0.6078	0.4310	0.2666	0.038*	
C17	0.48649 (18)	0.1574 (3)	0.25668 (5)	0.0298 (3)	
C18	0.37223 (19)	0.0579 (3)	0.22656 (6)	0.0291 (3)	
H18	0.3380	−0.0784	0.2340	0.035*	
C19	0.30745 (17)	0.1542 (2)	0.18583 (5)	0.0253 (3)	
H19	0.2308	0.0838	0.1652	0.030*	
C20	0.5594 (2)	0.0488 (3)	0.30016 (6)	0.0441 (5)	
H20A	0.5242	0.1161	0.3275	0.066*	
H20B	0.6729	0.0591	0.3020	0.066*	
H20C	0.5287	−0.0994	0.2992	0.066*	

### Atomic displacement parameters (Å^2^)

**Table:**

	*U*^11^	*U*^22^	*U*^33^	*U*^12^	*U*^13^	*U*^23^
S1	0.01901 (17)	0.0225 (2)	0.02499 (19)	−0.00027 (13)	0.00374 (13)	0.00294 (13)
O1	0.0367 (6)	0.0263 (6)	0.0254 (5)	−0.0045 (5)	0.0102 (4)	−0.0036 (4)
O2	0.0212 (5)	0.0360 (7)	0.0345 (6)	−0.0048 (5)	0.0009 (4)	0.0022 (5)
O3	0.0270 (5)	0.0233 (6)	0.0403 (6)	0.0038 (4)	0.0093 (5)	0.0044 (5)
C1	0.0227 (6)	0.0237 (7)	0.0195 (6)	−0.0006 (5)	0.0028 (5)	0.0026 (5)
C2	0.0217 (6)	0.0232 (7)	0.0193 (6)	0.0012 (5)	0.0031 (5)	0.0034 (5)
C3	0.0210 (6)	0.0231 (7)	0.0210 (6)	0.0007 (5)	0.0014 (5)	0.0047 (5)
C4	0.0240 (7)	0.0236 (7)	0.0266 (7)	0.0005 (6)	0.0037 (5)	0.0011 (6)
C5	0.0308 (8)	0.0261 (8)	0.0313 (8)	−0.0006 (6)	0.0030 (6)	−0.0022 (6)
C6	0.0299 (8)	0.0312 (9)	0.0375 (9)	−0.0099 (7)	0.0004 (6)	−0.0011 (7)
C7	0.0241 (7)	0.0358 (9)	0.0352 (8)	−0.0076 (6)	0.0048 (6)	0.0033 (7)
C8	0.0220 (6)	0.0291 (8)	0.0249 (7)	−0.0004 (6)	0.0026 (5)	0.0041 (6)
C9	0.0257 (7)	0.0366 (9)	0.0309 (8)	0.0011 (7)	0.0108 (6)	0.0043 (7)
C10	0.0331 (8)	0.0327 (9)	0.0286 (8)	0.0022 (7)	0.0124 (6)	0.0000 (6)
C11	0.0301 (7)	0.0234 (7)	0.0225 (7)	−0.0016 (6)	0.0056 (6)	0.0014 (6)
C12	0.0316 (7)	0.0256 (8)	0.0210 (7)	−0.0037 (6)	0.0042 (5)	0.0015 (6)
C13	0.0430 (9)	0.0297 (9)	0.0326 (8)	−0.0117 (7)	0.0066 (7)	−0.0041 (7)
C14	0.0233 (6)	0.0215 (7)	0.0216 (6)	0.0020 (5)	0.0061 (5)	0.0003 (5)
C15	0.0312 (7)	0.0259 (8)	0.0268 (7)	−0.0050 (6)	0.0048 (6)	−0.0002 (6)
C16	0.0334 (8)	0.0370 (9)	0.0250 (7)	−0.0034 (7)	0.0007 (6)	−0.0018 (7)
C17	0.0316 (8)	0.0362 (9)	0.0227 (7)	0.0050 (7)	0.0080 (6)	0.0045 (6)
C18	0.0339 (8)	0.0253 (8)	0.0297 (8)	0.0003 (6)	0.0097 (6)	0.0033 (6)
C19	0.0274 (7)	0.0231 (7)	0.0262 (7)	−0.0024 (6)	0.0069 (6)	0.0000 (6)
C20	0.0469 (10)	0.0543 (12)	0.0306 (9)	0.0023 (9)	0.0021 (8)	0.0138 (8)

### Geometric parameters (Å, º)

**Table:**

S1—O2	1.4354 (11)	C9—H9	0.9500
S1—O3	1.4366 (12)	C10—C11	1.400 (2)
S1—C1	1.7450 (15)	C10—H10	0.9500
S1—C14	1.7611 (15)	C12—C13	1.483 (2)
O1—C12	1.3562 (18)	C13—H13A	0.9800
O1—C11	1.3700 (18)	C13—H13B	0.9800
C1—C12	1.369 (2)	C13—H13C	0.9800
C1—C2	1.4632 (19)	C14—C19	1.390 (2)
C2—C11	1.381 (2)	C14—C15	1.390 (2)
C2—C3	1.436 (2)	C15—C16	1.386 (2)
C3—C4	1.413 (2)	C15—H15	0.9500
C3—C8	1.4293 (19)	C16—C17	1.394 (2)
C4—C5	1.370 (2)	C16—H16	0.9500
C4—H4	0.9500	C17—C18	1.391 (2)
C5—C6	1.405 (2)	C17—C20	1.506 (2)
C5—H5	0.9500	C18—C19	1.386 (2)
C6—C7	1.363 (2)	C18—H18	0.9500
C6—H6	0.9500	C19—H19	0.9500
C7—C8	1.414 (2)	C20—H20A	0.9800
C7—H7	0.9500	C20—H20B	0.9800
C8—C9	1.424 (2)	C20—H20C	0.9800
C9—C10	1.355 (2)		
			
O2—S1—O3	118.39 (7)	O1—C11—C2	111.64 (13)
O2—S1—C1	107.60 (7)	O1—C11—C10	122.26 (14)
O3—S1—C1	109.60 (7)	C2—C11—C10	126.08 (15)
O2—S1—C14	107.55 (7)	O1—C12—C1	110.07 (13)
O3—S1—C14	108.36 (7)	O1—C12—C13	113.98 (13)
C1—S1—C14	104.45 (7)	C1—C12—C13	135.95 (15)
C12—O1—C11	107.34 (11)	C12—C13—H13A	109.5
C12—C1—C2	107.44 (13)	C12—C13—H13B	109.5
C12—C1—S1	120.67 (11)	H13A—C13—H13B	109.5
C2—C1—S1	131.67 (11)	C12—C13—H13C	109.5
C11—C2—C3	117.89 (13)	H13A—C13—H13C	109.5
C11—C2—C1	103.51 (13)	H13B—C13—H13C	109.5
C3—C2—C1	138.60 (13)	C19—C14—C15	120.70 (14)
C4—C3—C8	117.86 (13)	C19—C14—S1	119.04 (11)
C4—C3—C2	125.43 (13)	C15—C14—S1	120.22 (12)
C8—C3—C2	116.70 (13)	C16—C15—C14	119.28 (15)
C5—C4—C3	121.38 (14)	C16—C15—H15	120.4
C5—C4—H4	119.3	C14—C15—H15	120.4
C3—C4—H4	119.3	C15—C16—C17	120.97 (15)
C4—C5—C6	120.75 (15)	C15—C16—H16	119.5
C4—C5—H5	119.6	C17—C16—H16	119.5
C6—C5—H5	119.6	C18—C17—C16	118.70 (14)
C7—C6—C5	119.30 (15)	C18—C17—C20	120.71 (16)
C7—C6—H6	120.4	C16—C17—C20	120.59 (16)
C5—C6—H6	120.4	C19—C18—C17	121.13 (15)
C6—C7—C8	121.82 (15)	C19—C18—H18	119.4
C6—C7—H7	119.1	C17—C18—H18	119.4
C8—C7—H7	119.1	C18—C19—C14	119.19 (14)
C7—C8—C9	119.84 (14)	C18—C19—H19	120.4
C7—C8—C3	118.89 (14)	C14—C19—H19	120.4
C9—C8—C3	121.26 (14)	C17—C20—H20A	109.5
C10—C9—C8	121.78 (14)	C17—C20—H20B	109.5
C10—C9—H9	119.1	H20A—C20—H20B	109.5
C8—C9—H9	119.1	C17—C20—H20C	109.5
C9—C10—C11	116.24 (15)	H20A—C20—H20C	109.5
C9—C10—H10	121.9	H20B—C20—H20C	109.5
C11—C10—H10	121.9		
			
O2—S1—C1—C12	−21.68 (14)	C12—O1—C11—C10	178.08 (15)
O3—S1—C1—C12	−151.66 (12)	C3—C2—C11—O1	−179.34 (12)
C14—S1—C1—C12	92.43 (13)	C1—C2—C11—O1	0.22 (16)
O2—S1—C1—C2	164.56 (13)	C3—C2—C11—C10	2.0 (2)
O3—S1—C1—C2	34.58 (16)	C1—C2—C11—C10	−178.40 (15)
C14—S1—C1—C2	−81.34 (15)	C9—C10—C11—O1	−178.71 (15)
C12—C1—C2—C11	0.24 (16)	C9—C10—C11—C2	−0.2 (2)
S1—C1—C2—C11	174.62 (12)	C11—O1—C12—C1	0.76 (16)
C12—C1—C2—C3	179.65 (17)	C11—O1—C12—C13	−178.63 (13)
S1—C1—C2—C3	−6.0 (3)	C2—C1—C12—O1	−0.62 (17)
C11—C2—C3—C4	177.50 (14)	S1—C1—C12—O1	−175.75 (10)
C1—C2—C3—C4	−1.8 (3)	C2—C1—C12—C13	178.57 (17)
C11—C2—C3—C8	−2.0 (2)	S1—C1—C12—C13	3.4 (3)
C1—C2—C3—C8	178.65 (16)	O2—S1—C14—C19	17.39 (14)
C8—C3—C4—C5	0.3 (2)	O3—S1—C14—C19	146.47 (11)
C2—C3—C4—C5	−179.20 (14)	C1—S1—C14—C19	−96.75 (12)
C3—C4—C5—C6	−0.3 (2)	O2—S1—C14—C15	−164.89 (12)
C4—C5—C6—C7	−0.1 (3)	O3—S1—C14—C15	−35.81 (14)
C5—C6—C7—C8	0.6 (3)	C1—S1—C14—C15	80.97 (13)
C6—C7—C8—C9	178.71 (15)	C19—C14—C15—C16	0.9 (2)
C6—C7—C8—C3	−0.6 (2)	S1—C14—C15—C16	−176.78 (12)
C4—C3—C8—C7	0.1 (2)	C14—C15—C16—C17	0.1 (2)
C2—C3—C8—C7	179.66 (13)	C15—C16—C17—C18	−1.6 (2)
C4—C3—C8—C9	−179.15 (14)	C15—C16—C17—C20	178.72 (16)
C2—C3—C8—C9	0.4 (2)	C16—C17—C18—C19	2.1 (2)
C7—C8—C9—C10	−177.80 (16)	C20—C17—C18—C19	−178.24 (15)
C3—C8—C9—C10	1.5 (2)	C17—C18—C19—C14	−1.1 (2)
C8—C9—C10—C11	−1.5 (2)	C15—C14—C19—C18	−0.4 (2)
C12—O1—C11—C2	−0.60 (17)	S1—C14—C19—C18	177.28 (11)

### Hydrogen-bond geometry (Å, º)

Cg is the centroid of the C14–C19 4-methylphenyl ring.

**Table:**

*D*—H···*A*	*D*—H	H···*A*	*D*···*A*	*D*—H···*A*
C13—H13*A*···O1^i^	0.98	2.54	3.503 (2)	168
C13—H13*C*···O3^ii^	0.98	2.49	3.440 (2)	164
C5—H5···*Cg*^iii^	0.95	2.80	3.648 (2)	149

Symmetry codes: (i) −*x*+1, −*y*, −*z*; (ii) *x*, *y*−1, *z*; (iii) *x*, *y*+1, *z*.
